# Supplementary material for: Single-Nucleotide Polymorphism in Genes Encoding G Protein Subunits GNB3 and GNAQ Increase the Risk of Cardiovascular Morbidity among Patients Undergoing Renal Replacement Therapy
Source: Int J Mol Sci. 2023 Oct 17;24(20):15260. doi: 10.3390/ijms242015260 (PMC10607787; doi:10.3390/ijms242015260)
Supplement: Supplementary file 1 [file ijms-24-15260-s001.zip › ijms-2637277-supplementary.pdf]

Supplementary Tables

Table S1. Occurrence of cardiovascular events and abnormal LVEDD increase among four different age groups of patients undergoing renal replacement therapy in relation to the three polymorphisms in genes coding for the G protein subunits *GNB3* c.825C>T, *GNAQ* -695/-694GC>TT, and *GNAS* c.393C>T. CI, confidence interval; LVEDD, left ventricular end-diastolic diameter; MACE, major adverse cardiovascular events; RR, relative risk.

				**RR (95% CI)**	***p* value**			**RR (95% CI)**	***p* value**			**RR (95% CI)**	***p* value**
**Age groups**													
**Age 18 - 39**	**All patients *n* = 120**	***GNB3* TT *n* = 14**	***GNB3* CT/CC *n* = 106**			***GNAQ* TT/TT *n* = 17**	***GNAQ* GC/TT/ GC/GC *n* = 103**			***GNAS* TT *n* = 36**	***GNAS* CT/CC *n* = 84**		
Cardiovascular event, n (%)	6 (5)	0 (0)	6 (6)	0.0 (0.00-4.1)	0.36	3 (18)	3 (3)	6.01 (1.5-24.0)	**0.01**	1 (3)	5 (6)	0.47 (0.1-2.8)	0.47
Cardiovascular event involving > 1 organ system, n (%)	1 (1)	0 (0)	1 (1)	0.0 (0.0-27.5)	0.72	1 (6)	0 (0)	Infinity (1.6-Infinity)	**0.01**	1 (3)	0 (0)	Infinity (0.6-Infinity)	0.13
Coronary artery stenosis requiring stent insertion, n (%)	3 (3)	0 (0)	3 (3)	0.0 (0.0-8.7)	0.52	2 (12)	1 (1)	12.10 (1.6-88.6)	**0.01**	1 (3)	2 (2)	1.17 (0.2-8.6)	0.90
MACE (cardiovasacular death, stroke, myocardial infarction), n (%)	4 (3)	0 (0)	4 (4)	0.0 (0.0-6.4)	0.46	2 (12)	2 (2)	6.06 (1.1-32.0)	**0.04**	1 (3)	3 (4)	0.78 (0.1-5.2)	0.82
Coronary artery disease diagnosed during RRT, n (%)	4 (3)	0 (0)	4 (4)	0.0 (0.0-6.4)	0.46	3 (18)	1 (1)	18.20 (2.7-122)	**0.0004**	1 (3)	3 (4)	0.78 (0.1-5.2)	0.82
Heart failure during RRT, n (%)	12 (10)	1 (7)	11 (10)	0.69 (0.1-3.4)	0.71	1 (6)	11 (11)	0.55 (0.1-2.8)	0.54	2 (6)	10 (12)	0.47 (0.1-1.7)	0.29
LVEDD male >56 mm/ female>51 mm(73/120), n (%)	17 (23)	3 (33)	14 (22)	1.52 (0.5-3.6)	0.45	4 (40)	13 (21)	1.30 (0.9-2.6)	0.18	5 (19)	12 (26)	0.75 (0.3-1.8)	0.54

**Age 40 - 49**	**All patients *n* = 94**	***GNB3* TT *n* = 14**	***GNB3* CT/CC *n* = 80**			***GNAQ* TT/TT *n* = 22**	***GNAQ* GC/TT/ GC/GC *n* = 72**			***GNAS* TT *n* = 23**	***GNAS* CT/CC *n* = 71**		
Cardiovascular event, n (%)	9 (10)	0 (0)	9 (11)	0.0 (0.0-2.0)	0.19	0 (0)	9 (13)	0.0 (0.0-1.2)	0.08	3 (13)	6 (8)	1.54 (0.4-5.1)	0.52
Cardiovascular event involving > 1 organ system, n (%)	2 (2)	0 (0)	2 (3)	0.0 (0.0-10.0)	0.55	0 (0)	2 (3)	0.0 (0.0-5.9)	0.43	1 (4)	1 (1)	3.09 (0.3-28.6)	0.40
Coronary artery stenosis requiring stent insertion, n (%)	3 (3)	0 (0)	3 (4)	0.0 (0.0-6.5)	0.46	0 (0)	3 (4)	0.0 (0.0-3.9)	0.33	0 (0)	3 (4)	0.0 (0.0-3.7)	0.32
MACE (cardiovasacular death, stroke, myocardial infarction), n (%)	6 (6)	0 (0)	6 (8)	0.0 (0.0-3.1)	0.29	0 (0)	6 (8)	0.0 (0.0-1.9)	0.16	2 (9)	4 (6)	1.54 (0.3-6.6)	0.60
Coronary artery disease diagnosed during RRT, n (%)	10 (11)	2 (14)	8 (10)	1.43 (0.4-5.0)	0.63	2 (9)	8 (11)	0.82 (0.2-3.0)	0.79	1 (4)	9 (13)	0.34 (0.1-1.9)	0.26
Heart failure during RRT, n (%)	14 (15)	2 (14)	12 (15)	0.95 (0.2-3.1)	0.95	1 (5)	13 (18)	0.25 (0.04-1.3)	0.12	4 (17)	10 (14)	1.23 (0.4-3.3)	0.70
LVEDD male >56 mm/ female > 51 mm(67/94), n (%)	14 (21)	2 (20)	12 (21)	0.95 (0.3-2.9)	0.94	3 (25)	11 (20)	1.25 (0.4-3.3)	0.70	4 (24)	10 (20)	1.18 (0.4-3.0)	0.76
**Age 50 - 59**	**All patients *n* = 136**	***GNB3* TT *n* = 11**	***GNB3* CT/CC *n* = 125**			***GNAQ* TT/TT *n* = 35**	***GNAQ* GC/TT/ GC/GC *n* = 101**			***GNAS* TT *n* = 41**	***GNAS* CT/CC *n* = 95**		
Cardiovascular event, n (%)	26 (19)	3 (27)	23 (18)	1.48 (0.5-3.5)	0.47	9 (26)	17 (17)	1.53 (0.7-3.0)	0.25	11 (27)	15 (16)	1.70 (0.9-3.3)	0.13

Cardiovascular event involving > 1 organ system, n (%)	5 (4)	0 (0)	5 (4)	0.0 (0.0-7.3)	0.50	3 (9)	2 (2)	4.33 (0.9-20.9)	0.07	1 (2)	4 (4)	0.58 (0.1-3.7)	0.61
Coronary artery stenosis requiring stent insertion, n (%)	13 (10)	0 (0)	13 (10)	0.0 (0.0-2.6)	0.26	8 (23)	5 (5)	4.62 (1.7-12.6)	**0.002**	5 (12)	8 (8)	1.45 (0.5-3.9)	0.49
MACE (cardiovasacular death, stroke, myocardial infarction), n (%)	13 (10)	1 (9)	12 (10)	0.95 (0.2-4.5)	0.96	4 (11)	9 (9)	1.28 (0.4-3.6)	0.66	5 (12)	8 (8)	1.45 (0.5-3.9)	0.49
Coronary artery disease diagnosed during RRT, n (%)	20 (15)	1 (9)	19 (15)	0.60 (0.1-2.7)	0.58	7 (20)	13 (13)	1.55 (0.7-3.4)	0.31	8 (20)	12 (13)	1.54 (0.7-3.4)	0.30
Heart failure during RRT, n (%)	23 (17)	1 (9)	22 (18)	0.52 (0.1-2.3)	0.47	6 (17)	17 (17)	1.02 (0.4-2.3)	0.97	9 (22)	14 (15)	1.49 (0.7-3.1)	0.30
LVEDD male >56 mm/ female > 51 mm(71/136), n (%)	19 (27)	4 (67)	15 (23)	2.89 (1.2-5.3)	**0.02**	3 (17)	16 (30)	0.55 (0.2-1.5)	0.26	5 (22)	14 (29)	0.76 (0.3-1.7)	0.51
**Age ≥ 60**	**All patients *n* = 104**	***GNB3* TT *n* = 6**	***GNB3* CT/CC *n* = 98**			***GNAQ* TT/TT *n* = 23**	***GNAQ* GC/TT/ GC/GC *n* = 81**			***GNAS* TT *n* = 18**	***GNAS* CT/CC *n* = 86**		
Cardiovascular event, n (%)	28 (27)	2 (33)	26 (26)	1.26 (0.4-3.0)	0.72	8 (35)	20 (25)	1.41 (0.7-2.6)	0.34	4 (22)	24 (28)	0.80 (0.3-1.8)	0.62
Cardiovascular event involving > 1 organ system, n (%)	6 (6)	1 (17)	5 (5)	3.27 (0.5-15.7)	0.24	2 (9)	4 (5)	1.76 (0.4-7.6)	0.50	0 (0)	6 (7)	0.0 (0.0-2.7)	0.25
Coronary artery stenosis requiring stent insertion, n (%)	15 (14)	1 (17)	14 (14)	1.17 (0.2-4.6)	0.87	4 (17)	11 (14)	1.28 (0.6-3.3)	0.65	1 (6)	14 (16)	0.34 (0.1-1.7)	0.24

MACE (cardiovasacular death, stroke, myocardial infarction), n (%)	13 (13)	0 (0)	13 (13)	0.0 (0.0-3.2)	0.34	4 (17)	9 (11)	1.57 (0.5-4.2)	0.42	0 (0)	13 (15)	0.0 (0.0-1.2)	0.08
Coronary artery disease diagnosed during RRT, n (%)	26 (25)	2 (33)	24 (24)	1.36 (0.4-3.2)	0.63	6 (26)	20 (25)	1.06 (0.5-2.2)	0.89	3 (17)	23 (28)	0.62 (0.2-1.6)	0.37
Heart failure during RRT, n (%)	19 (18)	2 (33)	17 (17)	1.92 (0.5-4.8)	0.33	5 (22)	14 (17)	1.26 (0.5-2.9)	0.63	2 (11)	17 (20)	0.56 (0.1-1.8)	0.39
LVEDD male >56 mm/ female > 51 mm(67/104), n (%)	28 (27)	1 (25)	27 (43)	0.58 (0.1-1.8)	0.48	7 (39)	21 (43)	0.91 (0.4-1.7)	0.77	3 (38)	25 (42)	0.90 (0.3-1.8)	0.79

Table S2. Occurrence of cardiovascular events and certain echocardiographic variables of the left ventricle among male and female patients undergoing renal replacement therapy in relation to the *GNAQ* -695/-694GC>TT and *GNAS* c.393C>T polymorphisms. CI, confidence interval; IVSd, interventricular septal end diastole; LVEDD, left ventricular end-diastolic diameter; LVPWd, left ventricular posterior wall end diastole; MACE, major adverse cardiovascular events; RR, relative risk; PAOD, peripheral artery.

	**Men**						**Women**						**Men**						**Women**					
			**RR (95% CI)**	**p value**	**p value after Bonferroni correction**	**p value after Benjamini & Hochberg correction**			**RR (95% CI)**	**p value**	**p value after Bonferroni correction**	**p value after Benjamini & Hochberg correction**			**RR (95% CI)**	**p value**	**p value after Bonferroni correction**	**p value after Benjamini & Hochberg correction**			**RR (95% CI)**	**p value**	**p value after Bonferroni correction**	**p value after Benjamini & Hochberg correction**
**Cardiovascular**	***GNAQ***	***GNAQ***					***GNAQ***	***GNAQ***					***GNAS***	***GNAS***					***GNAS***	***GNAS***				

**events during dialysis**	**TT/TT *n* = 52**	**GC/TT/GC/GC *n* = 234**					**TT/TT *n* = 45**	**GC/TT/GC/GC *n* = 123**					**TT *n* = 78**	**CT/CC *n* = 208**					**TT *n* = 40**	**CT/CC *n* = 128**				
Cardiovascular event, *n* (%)	11 (21)	40 (17)	1.24 (0.7-2.2)	0.55	1.0	0.94	9 (20)	9 (7)	2.73 (1.2-6.3)	**0.02**	0.32	0.11	14 (18)	37 (18)	1.01 (0.6-1.7)	0.98	1.0	0.99	5 (13)	13 (10)	1.23 (0.5-3.1)	0.68	1.0	0.85
Cardiovascular event involving > 1 organ system, n (%)	3 (6)	7 (3)	1.93 (0.6-6.5)	0.32	1.0	0.94	3 (7)	1 (1)	8.20 (1.2-56.3)	**0.03**	0.47	0.13	2 (3)	8 (4)	0.67 (0.2-2.7)	0.60	1.0	0.94	1 (3)	3 (2)	1.07 (0.2-7.2)	0.96	1.0	0.96
Acute PAOD, n (%)	2 (4)	11 (5)	0.82 (0.2-3.1)	0.79	1.0	0.94	4 (9)	1 (1)	10.90 (1.7-71.5)	**0.006**	0.12	**0.05**	4 (5)	9 (4)	1.19 (0.4-3.5)	0.77	1.0	0.94	2 (5)	3 (2)	2.13 (0.4-10.2)	0.39	1.0	0.73
Transient ischemic attack, n (%)	0 (0)	1 (0.4)	0.0 (0.0-17.0)	0.64	1.0	0.94	1 (2)	2 (2)	1.37 (0.2-10.1)	0.80	1.0	0.85	0 (0)	1 (0.5)	0.0 (0.0-10.1)	0.54	1.0	0.94	2 (5)	1 (1)	6.40 (0.9-47.9)	0.08	1.0	0.45
Stroke, n (%)	2 (4)	7 (3)	1.30 (0.3-5.2)	0.75	1.0	0.94	1 (2)	3 (2)	0.91 (0.1-6.1)	0.94	1.0	0.94	3 (4)	6 (3)	1.33 (0.4-4.7)	0.68	1.0	0.94	1 (3)	3 (2)	1.07 (0.2-7.2)	0.96	1.0	0.96

Carotid artery stenosis, n (%)	0 (0)	3 (1)	0.0 (0.0-5.6)	0.41	1.0	0.94	0 (0)	1 (1)	0.0 (0.0-10.3)	0.54	1.0	0.74	0 (0)	3 (1)	0.0 (0.0-3.3)	0.29	1.0	0.94	1 (3)	0 (0)	Infinity (0.8-Infinity)	0.07	1.0	0.45
Myocardial infarction, n (%)	4 (8)	15 (6)	1.20 (0.4-3.2)	0.74	1.0	0.94	3 (7)	3 (2)	2.73 (0.6-11.4)	0.19	1.0	0.40	4 (5)	15 (7)	0.71 (0.3-2.0)	0.53	1.0	0.94	1 (3)	5 (4)	0.64 (0.1-3.9)	0.68	1.0	0.85
Coronary artery stenosis requiring stent insertion, n (%)	9 (17)	18 (8)	2.30 (1.1-4.6)	**0.03**	0.54	0.51	5 (11)	2 (2)	6.83 (1.6-29.7)	**0.006**	0.12	**0.05**	6 (8)	21 (10)	0.76 (0.3-1.7)	0.54	1.0	0.94	1 (3)	6 (5)	0.53 (0.1-3.2)	0.55	1.0	0.85
Heart valve intervention, n (%)	3 (6)	10 (4)	1.35 (0.4-4.3)	0.64	1.0	0.94	1 (2)	2 (2)	1.37 (0.2-10.1)	0.80	1.0	0.85	6 (8)	7 (3)	2.29 (0.8-6.3)	0.12	1.0	0.94	1 (3)	2 (2)	1.60 (0.2-11.8)	0.70	1.0	0.85
Coronary artery bypass grafting, n (%)	3 (6)	9 (4)	1.50 (0.5-4.9)	0.53	1.0	0.94	0 (0)	2 (2)	0.0 (0.0-5.1)	0.39	1.0	0.66	5 (6)	7 (3)	1.90 (0.7-5.5)	0.25	1.0	0.94	0 (0)	2 (2)	0.0 (0.0-6.0)	0.43	1.0	0.73
MACE (cardiovascular death, stroke, myocardial	5 (10)	21 (9)	1.07 (0.4-2.6)	0.88	1.0	0.94	5 (11)	5 (4)	2.73 (0.9-8.4)	0.09	1.0	0.22	6 (7)	20 (10)	0.80 (0.4-1.8)	0.61	1.0	0.94	2 (5)	8 (6)	0.80 (0.2-3.1)	0.77	1.0	0.87

infarction), n (%)																								
**New onset of chronic cardiovascular disease during dialysis**																								
Coronary artery disease, n (%)	11 (21)	35 (15)	1.41 (0.8-2.5)	0.27	1.0	0.94	7 (16)	7 (6)	2.73 (1.0-7.0)	**0.04**	0.69	0.14	11 (14)	35 (17)	0.84 (0.5-1.5)	0.58	1.0	0.94	2 (5)	12 (9)	0.53 (0.1-2.0)	0.38	1.0	0.73
Coronary artery disease of ≥2 vessels, n (%)	5 (10)	19 (8)	1.18 (0.5-2.9)	0.73	1.0	0.94	3 (7)	2 (2)	4.10 (0.9-19.9)	0.09	1.0	0.22	7 (9)	17 (8)	1.10 (0.5-2.5)	0.83	1.0	0.94	0 (0)	5 (4)	0.0 (0.0-2.3)	0.20	1.0	0.57
Heart failure, n (%)	10 (19)	42 (18)	1.07 (0.6-1.9)	0.83	1.0	0.94	3 (7)	13 (11)	0.63 (0.2-1.9)	0.45	1.0	0.70	16 (21)	36 (17)	1.19 (0.7-2.0)	0.53	1.0	0.94	1 (3)	15 (12)	0.21 (0.03-1.2)	0.08	1.0	0.45
**Echocardiographic parameters of left ventricle**																								

LVEDD male >56 mm/ female > 51 mm(278/454), n (%)	7 (25)	42 (29)	0.86 (0.4-1.6)	0.66	1.0	0.94	10 (33)	19 (25)	1.33 (0.7-2.4)	0.39	1.0	0.66	13 (27)	36 (29)	0.91 (0.5-1.5)	0.72	1.0	0.94	4 (16)	25 (31)	0.52 (0.2-1.2)	0.15	1.0	0.51
IVSd male > 12 mm/female > 11 mm (299/454), n (%)	20 (61)	78 (48)	1.25 (0.9-1.7)	0.20	1.0	0.94	13 (50)	35 (44)	1.13 (0.7-1.7)	0.61	1.0	0.74	22 (44)	76 (54)	0.77 (0.5-1.1)	0.12	1.0	0.94	15 (54)	33 (43)	1.25 (0.8-1.9)	0.33	1.0	0.73
LVPWd male 12 mm/ female > 12mm (208/454), n (%)	10 (43)	48 (44)	1.0 (0.6-1.6)	0.99	1.0	0.99	3 (17)	13 (23)	0.73 (0.2-2.0)	0.58	1.0	0.74	17 (44)	41 (44)	1.0 (0.6-1.5)	1.0	1.0	0.99	2 (10)	14 (26)	0.37 (0.1-1.2)	0.12	1.0	0.51
